# The antimicrobial peptide PFR induces necroptosis mediated by ER stress and elevated cytoplasmic calcium and mitochondrial ROS levels: cooperation with Ara-C to act against acute myeloid leukemia

**DOI:** 10.1038/s41392-019-0073-6

**Published:** 2019-10-04

**Authors:** Yudie Lv, Gang Shao, Qiyu Zhang, Xi Wang, Yueming Meng, Lingfei Wang, Feiyan Huang, Tianxin Yang, Yuanting Jin, Caiyun Fu

**Affiliations:** 1 College of Life Sciences and Medicine, Zhejiang Sci-Tech University, 310018 Hangzhou, China; 2 College of Life Sciences, China Jiliang University, 310018 Hangzhou, China; 3 Department of Oncology, The 903rd Hospital of PLA, 310013 Hangzhou, China; 4 Clinical laboratory, Zhejiang Provincial Hospital of TCM, 310006 Hangzhou, China; 5 Department of Hematology, Zhejiang Province People’s Hospital, 310014 Hangzhou, China

**Keywords:** Haematological cancer, Drug development

**Dear Editor,**

Antimicrobial peptides (AMPs) are an ancient class of short polypeptides present in a large number of species in nature with a variety of functions.^1^ PFR (PFWRIRIRR-NH_2_) is one kind of AMP identified among the derivatives of lactoferrin.^2^ Our previous results showed that PFR inhibited the proliferation of human acute myeloid leukemia (AML) HL60 cells potentially without toxicity against normal cells. In addition, PFR induced necrosis by membrane disruption detected using scanning electron microscopy.^3^ However, the underlying mechanisms of these effects are not clearly understood.

To investigate the mechanisms involved in necrosis^4^ induced by PFR in HL60 cells (Fig. S1a–d), we found that 5(6)-FAM was taken up by HL60 cells after PFR treatment in a time-dependent manner (Fig. 1a), indicating that PFR induced the formation of permeable pores with open diameters of at least the molecular size of 5(6)-FAM (≈1 nm). In addition, levels of phosphorylated RIP1, RIP3, and MLKL were increased significantly after PFR treatment (Figs. 1b and S1e), indicating that necroptosis had occurred. Furthermore, necrostatin-1 (Nec-1), a specific inhibitor of necroptosis, significantly reduced propidium iodide (PI) uptake induced by PFR (Fig. 1c).

**Fig. 1:**
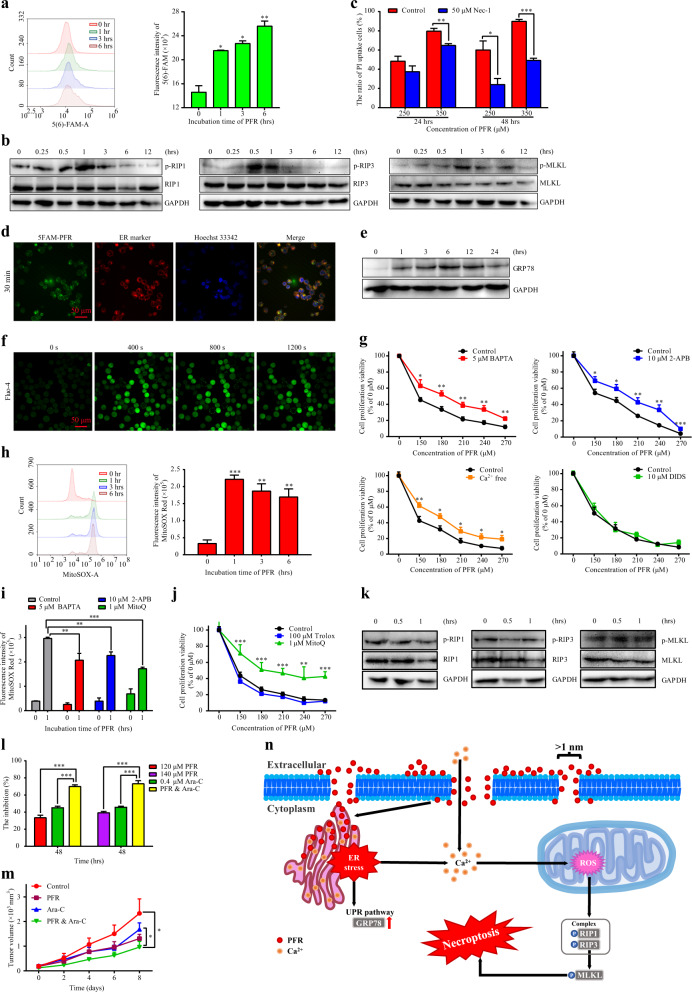
PFR induces necroptosis through ER stress and elevated cytoplasmic calcium and mitochondrial ROS levels and cooperates with Ara-C to act against acute myeloid leukemia. Detailed explanations for all subfigures are given in the Supplementary Information

We further synthesized green fluorescent 5-FAM-PFR and traced its dynamic location for up to 6 h (Fig. S2a). The dynamic distribution of PFR on the cytomembrane (~3–10 min) and endoplasmic reticulum (ER) (after 30 min) was clearly indicated by green and bright yellow fluorescence, respectively (Figs. 1d and S2a). The unexpected localization of PFR on the ER prompted us to detect whether PFR induces ER stress because of the fact that ER stress is involved in cell death.^5,6^ The expression level of the classic ER stress marker GRP78 was increased significantly after PFR treatment (Figs. 1e and S2b).

That PFR can target the ER to induce ER stress was completely unexpected. The ER plays an essential role in regulating Ca^2+^ homeostasis.^7^ Thus, we monitored intracellular calcium mobilization in response to PFR treatment. PFR caused a rapid and consistent increase in cytosolic calcium (observed by Fluo-4 staining) followed by a delayed and moderate increase in mitochondrial calcium concentration (observed by Rhod-2 staining) in a dose-dependent manner (Figs. 1f and S3a). The calcium chelator BAPTA; 2-APB, which inhibits the IP3R ER calcium channel; and culture in calcium-free medium could reduce cell death induced by PFR treatment (Fig. 1g). This effect was not observed following treatment with DIDS, which inhibits the voltage-dependent anion channel type 1 calcium channel on the outer mitochondrial membrane, indicating that elevated cytoplasmic calcium from both the influx of extracellular Ca^2+^ and release of intracellular ER Ca^2+^ induced by ER stress, but not mitochondrial calcium, mediates the cytotoxicity of PFR in HL60 cells. As ROS production is the executioner and mediator of necroptosis,^8^ cytosolic ROS were decreased significantly (Fig. S3b), while mitochondrial ROS were increased significantly after PFR treatment (Fig. 1h). Moreover, both BAPTA and 2-APB significantly decreased mitochondrial ROS production induced by PFR treatment (Fig. 1i), indicating that elevated cytoplasmic calcium contributes to elevated mitochondrial ROS. The mitochondrial-targeted antioxidant MitoQ both decreased elevated mitochondrial ROS levels (Fig. 1i) and increased cell proliferation and viability (Fig. 1j) in the presence of PFR, while the cytosolic antioxidant Trolox had no obvious effect on cell proliferation and viability in the presence of PFR (Fig. 1j). Meanwhile, the PFR-induced increase in phosphorylated RIP1, RIP3, and MLKL levels (Figs. 1b and S1e) was blocked by pretreatment with MitoQ (Figs. 1k and S3c), indicating that the elevation of mitochondrial ROS is a major mechanism of necroptosis by RIP1-RIP3-MLKL activation in response to PFR treatment.

Cytosine arabinoside (Ara-C) is a common drug used in the treatment of AML with the drawbacks of drug resistance and drug-related toxicity.^9^ Cooperation between PFR and Ara-C (Fig. 1l) increased the number of necrotic cells (Fig. S4a). Similarly, PI uptake (Fig. S4b) and LDH release (Fig. S4c) were increased significantly in the group treated with both PFR and Ara-C. Combined treatment with PFR and Ara-C also significantly inhibited the growth of tumors in the HL60 xenograft mouse model (Figs. S5a–c and 1m). In addition, no serious side effects and no difference in weight gain (Fig. S5d) were observed in the combined group, and no toxicity was detected in the liver (Fig. S5e) and kidney (Fig. S5f).

In summary, we found a novel mechanism by which PFR induces necroptosis through ER stress, elevated cytoplasmic calcium, and mitochondrial ROS (Fig. 1n). Furthermore, PFR can also cooperate with Ara-C to enhance the efficacy of Ara-C in vitro and in vivo. The novel molecular mechanisms of PFR used to treat AML and the efficacy of cooperation between PFR and Ara-C may provide new insights into the molecular mechanisms of AMP and a new therapeutic option to treat human AML.

## Supplementary information

Supplementary Information
