# Supplementary material for: The antimicrobial peptide PFR induces necroptosis mediated by ER stress and elevated cytoplasmic calcium and mitochondrial ROS levels: cooperation with Ara-C to act against acute myeloid leukemia
Source: Signal Transduct Target Ther. 2019 Oct 4;4:38. doi: 10.1038/s41392-019-0073-6 (PMC6799817; doi:10.1038/s41392-019-0073-6)
Supplement: Supplementary file 1 — Supplementary Information [file 41392_2019_73_MOESM1_ESM.doc]

Supplementary Materials for

**The antimicrobial peptide PFR induces** **necroptosis mediated by** **ER stress and elevated cytoplasmic calcium and mitochondrial ROS levels: cooperation with Ara-C to act against acute myeloid leukemia**

Yudie Lv, Gang Shao, Qiyu Zhang, Xi Wang, Yueming Meng, Lingfei Wang, Feiyan Huang, Tianxin Yang, Yuanting Jin, Caiyun Fu

*Correspondence e-mail: [fucy03@zstu.edu.cn](mailto:fucy03@zstu.edu.cn) to Caiyun Fu

**This file includes:**

Figure legend for Fig. 1 2

Supplemental Figures 4

Fig. S1 PFR induces necroptosis in HL60 cells 4

Fig. S2 PFR targets on ER to induce ER stress in HL60 cells 5

Fig. S3 PFR induces necroptosis in HL60 cells mediated by elevated cytoplasmic calcium and mitochondria ROS 6

Fig. S4 PFR cooperation with Ara-C enhance the efficacy of Ara-C *in vitro* 7

Fig. S5 PFR cooperation with Ara-C inhibit the growth of HL60 cells *in vivo* 8

**Fig. 1 PFR induces necroptosis through ER stress, elevated cytoplasmic calcium and mitochondrial ROS, as well as cooperates with Ara-C against acute myeloid leukemia.** (a) 5(6)-FAM (2 μM) was uptake by HL60 cells after 250 μM PFR treatment at indicated time points detected by flow cytometry. Values represent means ± SEM (n = 3). **P* < 0.05, ***P* < 0.01, compared with the control group. (b) Western blotting of necroptosis related proteins in HL60 cells treated with 150 μM PFR. The statistical results were shown in Fig. S1e. (c) A specific inhibitor of necroptosis, Nec-1, markedly inhibited the PI uptake induced by PFR treatment at 24 and 48 hours. Values represent means ± SEM (n = 3). **P*< 0.05, ***P* < 0.01, ****P* < 0.001, compared with the control group. (d) The localization of 5FAM-PFR (150 μM) was traced dynamically using laser scanning confocal microscope at 30 min. The total images for 6 hours were shown in Fig. S2a. Scale bar represents 50 μm. (e) Western blotting of ER stress marker GRP78 treated with 150 μM PFR in HL60 cells at indicated time points. The statistical results were shown in Fig. S2b. (f) Representative images of cytosolic calcium indicator Fluo-4 AM staining after PFR treatment (350 μM). The representative images and quantitative results of cytosolic and mitochondrial calcium were shown in Fig. S3a. (g) The cell proliferation viability was calculated as the percentage of living cells in the PFR treatment group relative to the control group at 24 hours. HL60 cells were pre-treated with BAPTA (5 μM), 2-APB (10 μM) or DIDS (10 μM) for 1 hour, or HL60 cells were cultured in the medium in the presence or the absence of Ca^2+^, and then treated with PFR at indicated concentrations. The living cells were counted by trypan blue exclusion. Values represent means ± SEM (n = 3). **P*< 0.05, ***P* < 0.01, ****P* < 0.001, compared with the control group at each corresponding dose. (h) The mitochondrial superoxide levels were measured by MitoSOX (5 μM) at 0, 1, 3 and 6 hours using flow cytometry after PFR (250 μM) treatment. Values represent means ± SEM (n = 3). ***P*< 0.01, ****P* < 0.001, compared with the control group. (i) HL60 cells were pre-treated with BAPTA (5 μM), 2-APB (10 μM) or MitoQ (1 μM) for 1 hour, and then treated with PFR (250 μM). The mitochondrial superoxide levels were measured by MitoSOX (5 μM) at 0 and 1 hour using flow cytometry. Values represent means ± SEM (n = 3). ***P*< 0.01, ****P* < 0.001, compared with the control group. (j) HL60 cells were pre-treated with Trolox (100 μM) or MitoQ (1 μM) for 1 hour and then treated with PFR at the indicated doses for 24 hours. The cell proliferation viability was calculated as the percentage of living cells in the PFR treatment group relative to each corresponding control group. The living cells were counted by trypan blue exclusion. ***P*< 0.01, ****P* < 0.001, compared with the control group at each corresponding dose. (k) HL60 cells were pre-treated with 1 μM MitoQ for 1 hour and then treated with PFR (150 μM). Western blotting of necroptosis related proteins RIP1, RIP3, and MLKL at indicated time points. The statistical results were shown in Fig. S3c. (l) Effect of PFR combined with Ara-C on the proliferation of HL60 cells detected by MTT assay. Values represent means ± SEM (n = 3). ****P* < 0.001, compared with the indicated group. (m) Tumor volume was measured in HL60 xenografts every two days. Values represent means ± SEM (n = 3 in each group). **P* < 0.05, compared with the indicated group. (n) Schematic of PFR to induce necroptosis via ER stress, elevated cytoplasmic calcium and mitochondria ROS.

**
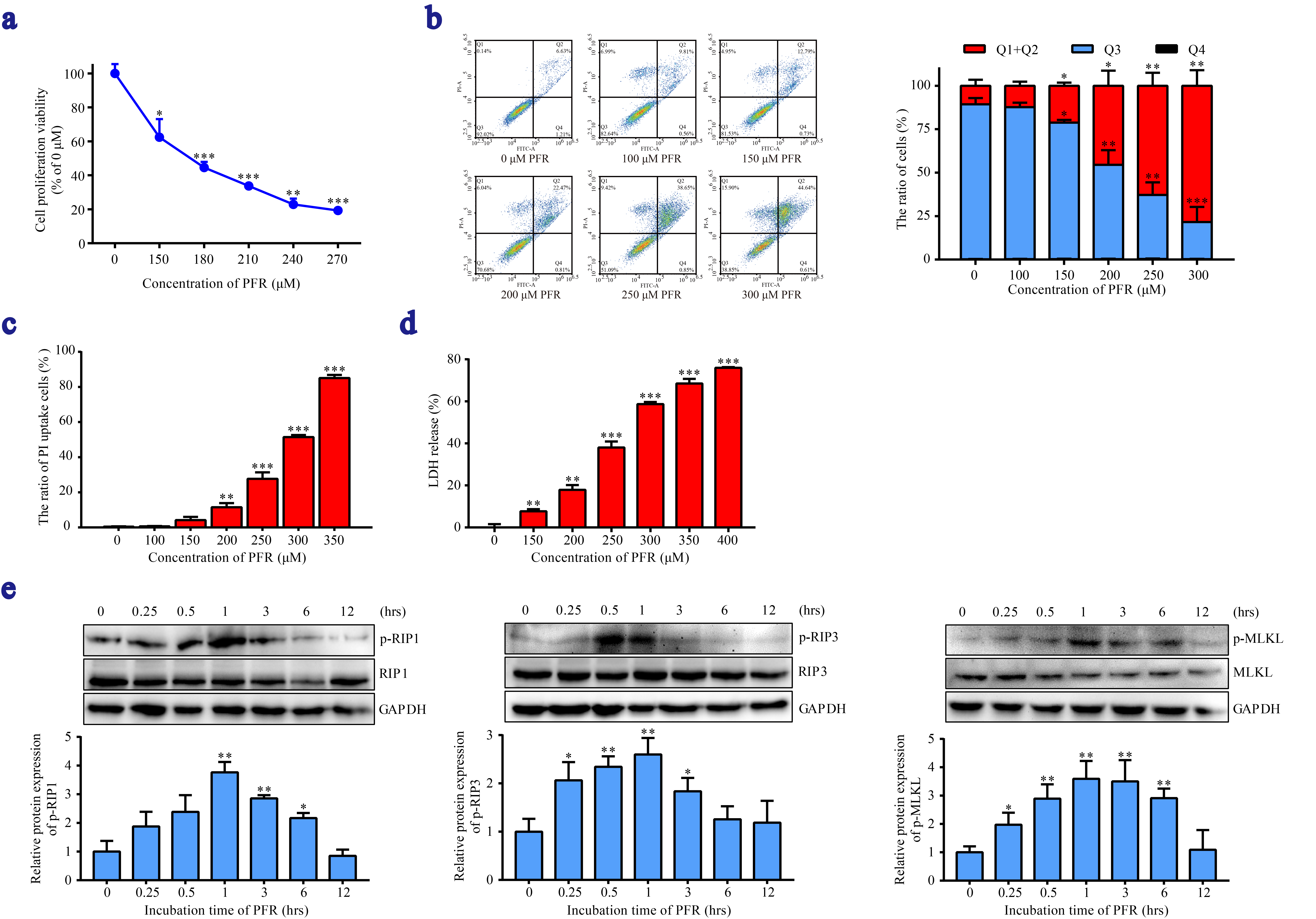
**

**Fig. S1 PFR induces necroptosis in HL60 cells.** (a) PFR inhibited the proliferation of HL60 cells in a dose-dependent manner for 24 hours. The living cells were counted by trypan blue exclusion. Values represent means ± SEM (n = 3). **P*< 0.05, ***P* < 0.01, ****P* < 0.001, compared with the control group. (b) Flow cytometry analysis of necrosis by Annexin-V-FITC/PI staining in HL60 cells after PFR treatment at indicated concentrations for 24 hours. Values represent means ± SEM (n = 3). **P*< 0.05, ***P* < 0.01, ****P* < 0.001, compared with the control group. (c and d) Treatment of HL60 cells with PFR resulted in a concentration-dependent increase in PI uptake for 24 hours (c) and LDH release for 6 hours (d). Values represent means ± SEM (n = 3). ***P* < 0.01, ****P* < 0.001, compared with the control group. (e) Western blotting of necroptosis related proteins in HL60 cells treated with 150 μM PFR at indicated time points. Values represent means ± SEM (n = 3). **P*< 0.05, ***P* < 0.01, compared with the control group.


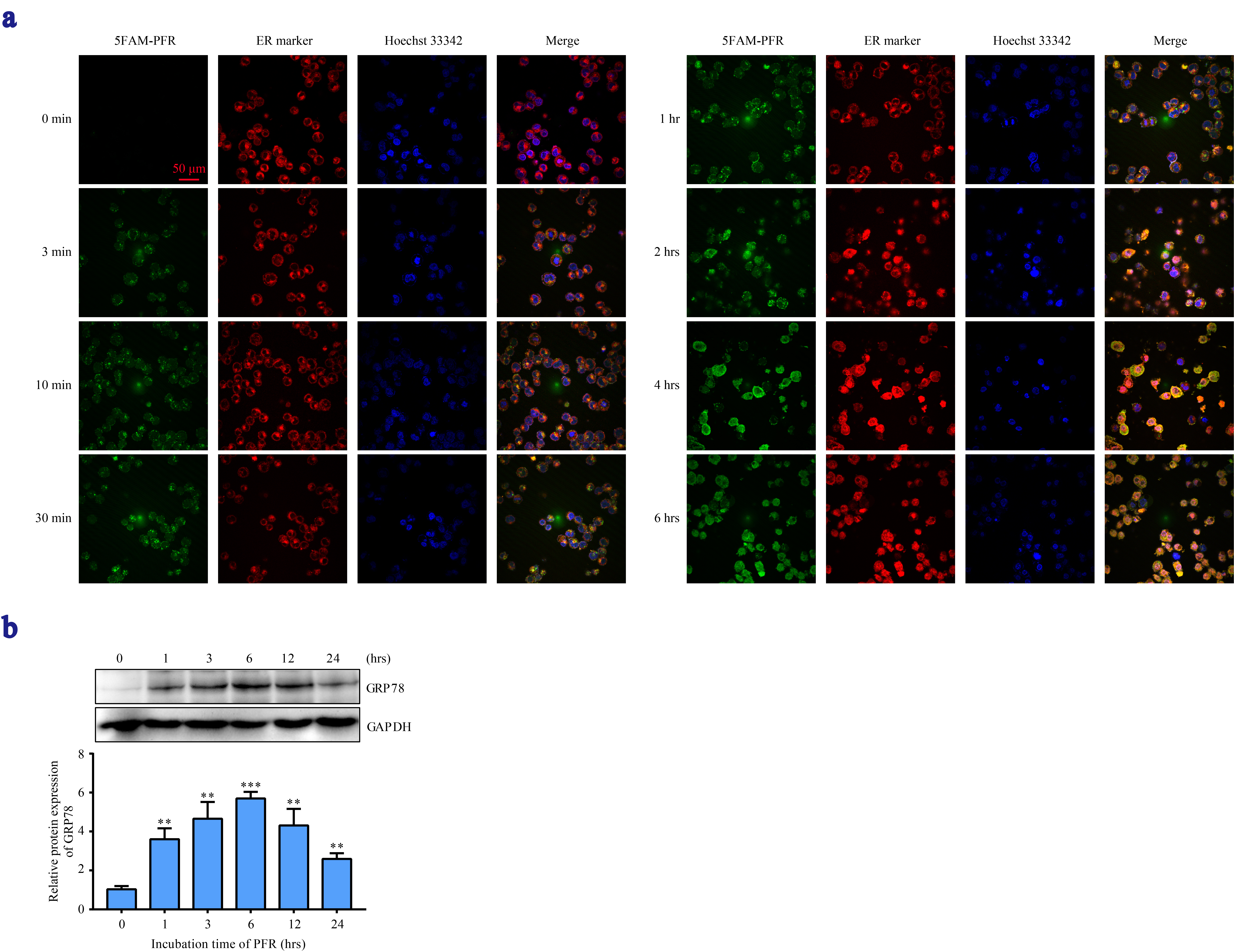


**Fig. S2** **PFR targets on ER to induce ER stress in HL60 cells.** (a) The localization of 5FAM-PFR (150 μM) was traced dynamically using laser scanning confocal microscope for up to 6 hours. Scale bar represents 50 μm. (b) Western blotting of ER stress marker GRP78 treated with 150 μM PFR in HL60 cells at indicated time points. Values represent means ± SEM (n = 3). ***P* < 0.01, ****P* < 0.001, compared with the control group.


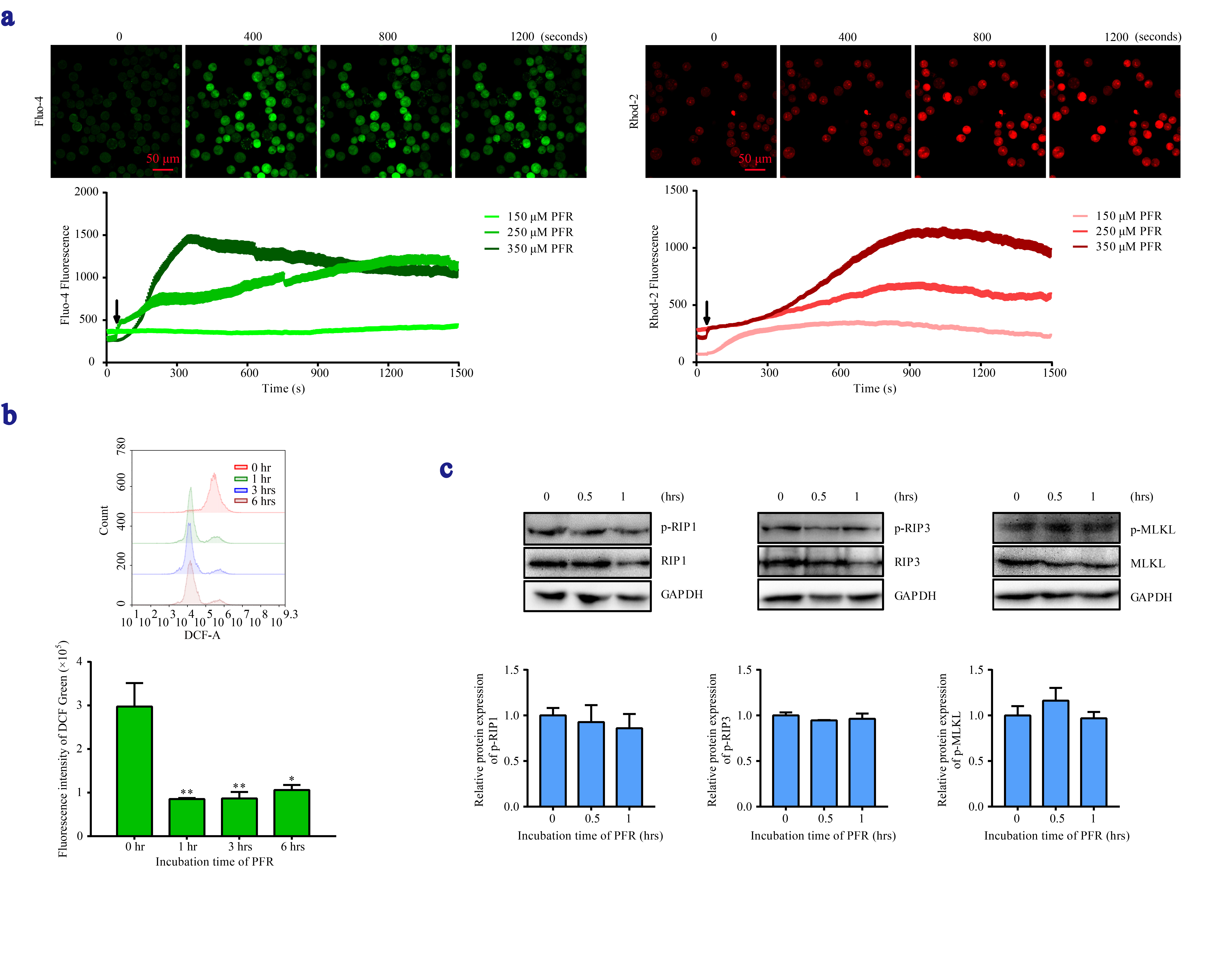


**Fig. S3 PFR induces necroptosis in HL60 cells mediated by elevated cytoplasmic calcium and mitochondria ROS.** (a) Upper: Representative images of cytosolic calcium indicator Fluo-4 AM staining and mitochondrial calcium indicator Rhod-2 AM staining in HL60 cells after PFR treatment (350 μM). Lower: Quantitative results of Fluo-4 AM and Rhod-2 AM fluorescence intensity in HL60 cells at indicated doses. Calcium concentrations were expressed as the average fluorescence intensity of 20 cells/field randomly from at least three fields at each time point. After the initial measurement for 50 seconds to determine the baseline fluorescence, PFR at 150, 250 and 350 μM, was added into the culture medium. Image acquisition continued for 1500 seconds after drug treatment. Arrow indicated the time point to add PFR. Scale bar represents 50 μm. (b) The cytosolic superoxide levels were measured by DCF (10 μM) at 0, 1, 3 and 6 hours using flow cytometry after PFR (250 μM) treatment. Values represent means ± SEM (n = 3). **P*< 0.05, ***P* < 0.01, compared with the control group. (c) HL60 cells were pre-treated with MitoQ (1 μM) for 1 hour and then treated with PFR (150 μM). Western blotting of necroptosis related proteins RIP1, RIP3, and MLKL at indicated time points. Values represent means ± SEM (n = 3).


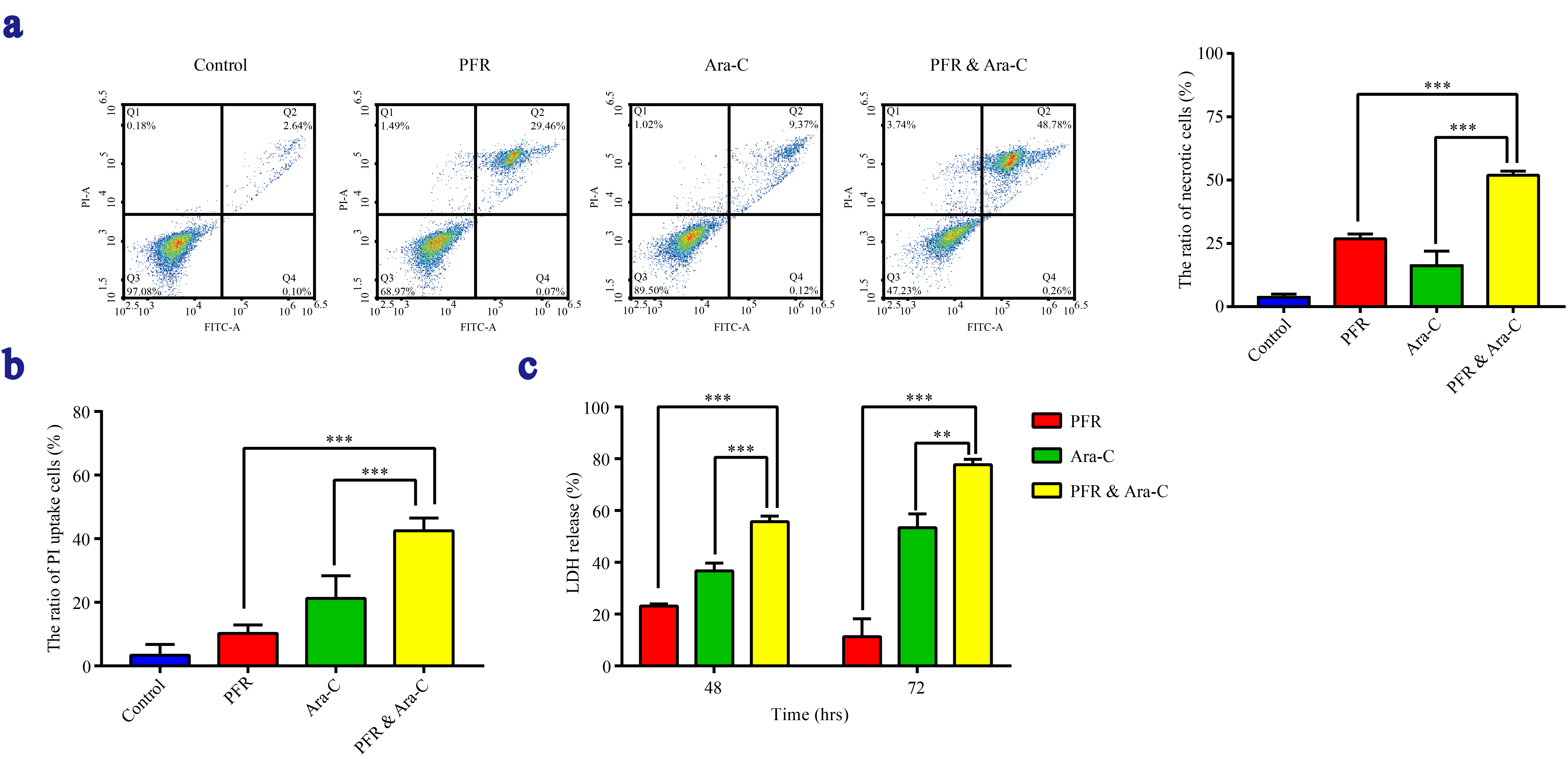


**Fig. S4 PFR cooperation with Ara-C enhance the efficacy of Ara-C *in vitro*.** (a) Annexin-V-FITC plus PI analysis of HL60 cells treated with PFR (120 μM) and Ara-C (1.8 μM). After 24 hours, the ratio of necrosis in PFRs combined with Ara-C group was increased significantly. ****P* < 0.001, compared with the indicated group. (b) PI uptake was detected at 24 hours with PFR (120 μM) and Ara-C (1 μM). *** *P* < 0.001, compared with the indicated group. (c) LDH release was detected at 48 hours and 72 hours with PFR (120 μM) and Ara-C (0.6 μM). ** *P* < 0.01, *** *P* < 0.001, compared with the indicated group.


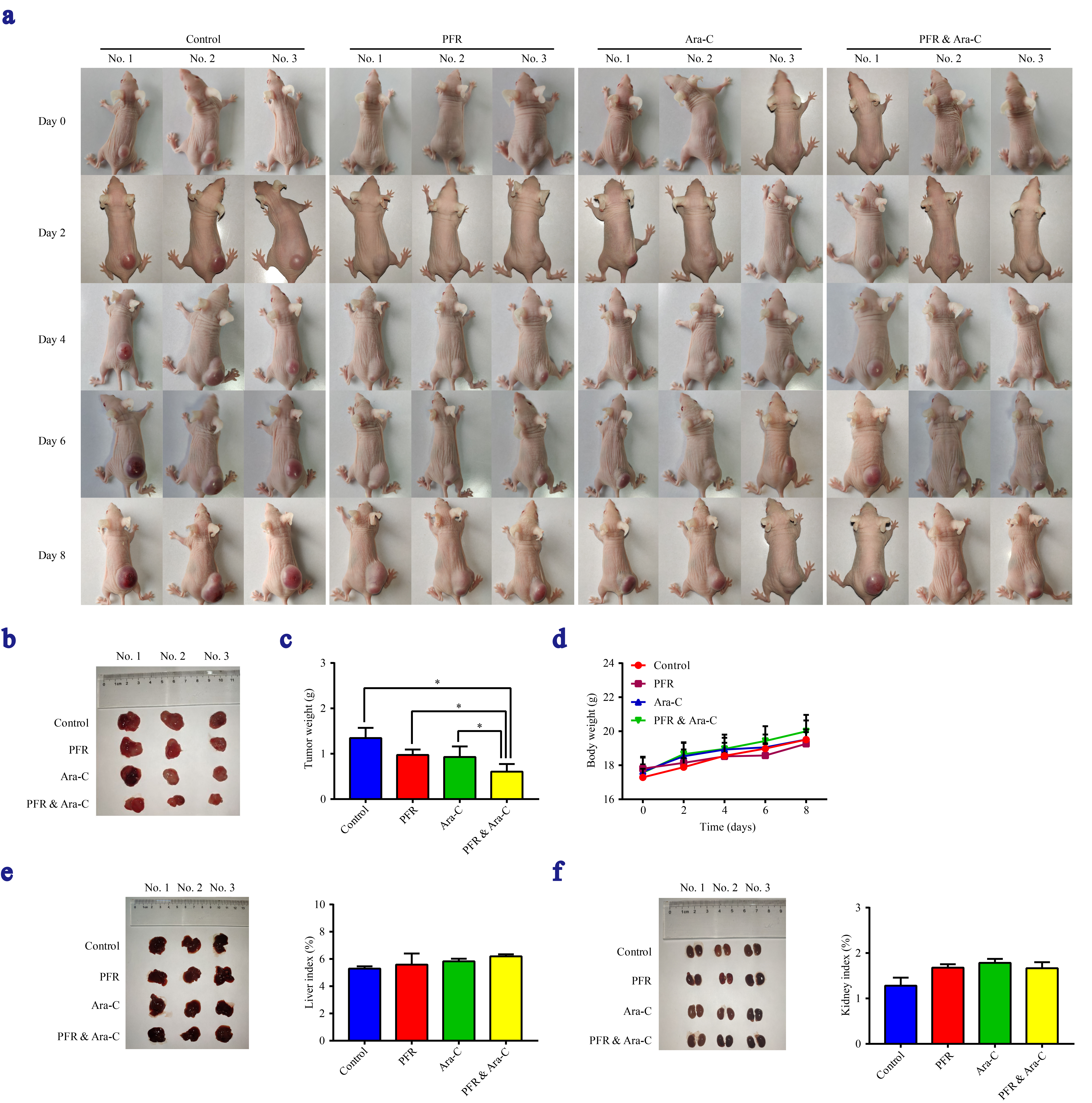


**Fig. S5** **PFR cooperation with Ara-C inhibit the growth of HL60 cells *in vivo*.** (a) The images of mice bearing HL60 tumors in each group photographed every two days. (b) The images of tumors excised from all mice on day 8. (c) Tumor weight after excised from all mice was calculated on day 8. Values represent means ± SEM (n = 3 in each group). **P* < 0.05, compared with the indicated group. (d) Body weight measured in HL60 tumor bearing mice every two days. Values represent means ± SEM (n = 3 in each group). (e and f) The images of liver (e) and kidney (f) excised from each mouse. The weight of liver or kidney divided by the weight of each corresponding mouse indicated the liver index or kidney index. Values represent means ± SEM (n = 3 in each group).
